# Mucinous Cancer of the Ovary: Overview and Current Status

**DOI:** 10.3390/diagnostics10010052

**Published:** 2020-01-19

**Authors:** Abdulaziz Babaier, Prafull Ghatage

**Affiliations:** 1 Department of Gynecologic Oncology, King Fahad Specialist Hospital, Dammam 32253, Saudi Arabia; 2 Department of Gynecologic Oncology, Tom Baker Cancer Centre, Calgary, AB T2N4N2, Canada; prafull.ghatage@albertahealthservices.ca

**Keywords:** mucinous ovarian carcinoma, metastatic mucinous carcinoma, genomic profile, surgery, chemotherapy, targeted therapy

## Abstract

Mucinous ovarian cancer (MOC) is a rare subtype of epithelial ovarian carcinoma (EOC). Whereas all EOC subtypes are addressed in the same way, MOC is a distinct entity. Appreciating the pathological features and genomic profile of MOC may result in the improvement in management and, hence, the prognosis. Distinguishing primary MOC from metastatic mucinous carcinoma can be challenging but is essential. Early-stage MOC carries an excellent prognosis, with advanced disease having a poor outcome. Surgical management plays an essential role in the early stage and in metastatic disease. Chemotherapy is usually administered for stage II MOC and beyond. The standard gynecology protocol is frequently used, but gastrointestinal regimens have also been administered. As MOC is associated with multiple molecular alterations, targeted therapy could be the answer to treat this disease.

## 1. Background

Ovarian cancer is the second most common gynecological malignancy, but the most lethal [1]. Epithelial ovarian cancer (EOC) is the most common histological type. EOC is classified, based on molecular and clinic-pathologic differences, into Type 1 tumors, which include low-grade serous carcinoma, endometrioid carcinoma, clear cell carcinoma, and mucinous ovarian carcinoma (MOC), and Type 2 tumors, which include high-grade serous carcinoma (HGSC) [2,3]. While HGSC is the most frequent histological subtype, mucinous carcinoma of the ovary is sporadic. MOC was believed to constitute around 12% of ovarian malignancies. However, recent estimations show the true incidence to be at around 3% [4,5]. The two main reasons for this drop in incidence are the identification criteria, which separate benign mucinous tumors from invasive mucinous carcinoma, and better recognition of clinical and pathological features to differentiate between primary mucinous carcinoma and metastatic carcinoma of the ovary [6].

It is clearly understood that MOC is a separate entity from all other EOCs. It has a distinct natural history, molecular profile, chemo-sensitivity, and prognosis in comparison to HGSC. A comprehensive report on the genomic profile of HGSC by the Cancer Genome Atlas Research Network in 2011 revealed a distinct mutation spectrum among high-grade serous tumors and opened the door for potential targeted therapies [7].

MOC is the most frequent histological subtype in women under the age of 40 [8]. The well-known risk factors for HGSC, such as nulliparity, early menarche, late menopause, lack of breastfeeding, *BRCA* (Breast Cancer Gene) mutation, are not associated with MOC. The only possible risk factor correlated with MOC is tobacco smoking [9]. Most HGSCs present at an advanced stage, while MOC is diagnosed as stage 1 in 80% of the cases [10]. Prognosis is better in early disease, but worse in the advanced stage, compared to HGSC, which is mainly due to inadequate response to platinum-based chemotherapy [11,12].

## 2. Histogenesis

Normal mucinous epithelium comprises three types of mucus-secreting cells, which line the stomach (gastric), endocervix (endocervical), and intestine (intestinal). The normal ovarian tissues do not include any of the mucin-secreting cells. There are multiple theories to explain the development of MOC:

- Adenoma carcinoma sequence “stepwise fashion”. The existence of mucinous cystadenoma and mucinous borderline components with carcinoma supports this theory. The carcinoma grows from benign epithelium to borderline tumor to invasive carcinoma. *KRAS* mutation occurs early in the process, while TP53 mutation and HER2 amplification occur later as they are exclusively detected in mucinous carcinoma [5,13,14,15].
- Germ cell origin is proposed by association with mature teratoma in 5% of cases and the universal existence of gastrointestinal-type cells, in addition to the gastrointestinal and pancreatico-biliary markers. However, most MOCs do not have any teratomatous components [3,16].
- Mucinous metaplasia of the ovarian surface epithelium or within the lining of cortical inclusion cysts [3,16].
- Strong association with endometriosis. They are usually endocervical-like or Mullerian mucinous tumors [3,16].
- Mucinous epithelium frequently presents with Brenner tumors. Mucinous carcinoma, mainly the intestinal type, may evolve from transitional cells or metaplasia at the fallopian tube-peritoneal junction [3,16].

## 3. Pathological Aspects

Around 80% of mucinous carcinomas of the ovary are metastatic, with approximately 80% of primary tumors being stage I. The most frequent primary sites that metastasize to the ovary are: 45% from the gastrointestinal tract, 20% from the pancreas, 18% from the cervix and endometrium, and 8% from the breast [17,18]. It is agreed that diagnosing primary MOC requires careful pathological assessment as it is histologically very similar to other mucinous carcinomas, especially colorectal carcinoma (CRC). Recognizing the microscopic features and understanding the immunohistochemistry (IHC) profile of MOC are essential to reach a definitive diagnosis, which results in delivering proper treatment and an accurate prognosis.

MOC is usually a heterogeneous tumor. It encompasses benign, borderline, and carcinoma components, which indicate a stepwise progression to carcinoma. The diagnosis of an invasive carcinoma requires the detection of stromal invasion of more than 5 mm or more than 10 mm^2^. Invasion less than these measurements is classified as “micro-invasion” with a borderline mucinous tumor. MOC is typically the intestinal type, but the endocervical type may develop infrequently [19,20,21]. According to the growth and invasion pattern, Lee and Schully classified MOC into expansile and infiltrative subtypes [22]. The expansile subtype has no destructive stromal invasion, but exhibits confluent or complex malignant glands (back to back glands) with or without minimal intervening stroma exceeding a 10 mm^2^ area or >3 mm each of two linear dimensions. The infiltrative type has stromal invasion in the form of glands, cell clusters, or individual cells, unsystematically infiltrating the stroma and often associated with a desmoplastic stromal reaction [20,21,22,23]. In 2014, the World Health Organization (WHO) adopted Lee and Schully’s classification for MOC.

Certain histological features are suggestive for metastatic mucinous carcinoma. In general, mucinous carcinomas are categorized into cystic and colloid type, based on intracellular or extracellular mucin localization. Ovarian and pancreatic cystic mucinous carcinomas contain a large amount of intracellular mucin (>50%) in at least 90% of tumor cells. On the other hand, colloid mucinous carcinomas arising from the gastrointestinal tract, lung, breast, and skin are associated with abundant extracellular mucin accounting for 50% or more tumor volume [6]. Seidman et al. proposed an algorithm based on tumor size and laterality to distinguish between MOC and metastatic mucinous carcinoma. Tumors that were ≥10 cm and unilateral were primary MOCs 82% of the time. Unilateral tumors <10 cm were metastatic 87% of the time. Bilateral tumors <10 cm were metastatic in 92% of cases and when bilateral and ≥10 cm they were metastatic in 95% of cases [4,24]. Therefore, the possibility of metastatic mucinous carcinoma should always be considered, even in the case of a unilateral tumor. Moreover, features that suggest that metastatic disease is more likely are [25,26,27]:

- Bilateral disease;
- Ovarian surface involvement;
- Extracellular mucin localization;
- Destructive stromal invasion;
- Nodular growth pattern;
- Hilar involvement;
- Vascular invasion;
- Signet ring cells;
- Extensive necrosis.

In addition to the microscopic features, IHC staining plays an essential role in distinguishing MOC from other possible diagnoses. MOC typically shares positive IHC patterns for CK20, CEA, Ca19-9, and CDX2 with metastatic CRC. Nevertheless, CK7 is mostly positive in MOC and negative in CRC. Table 1 summarizes the IHC profile for MOC and metastatic mucinous carcinoma [11,19,28,29]. The standard IHC profile for MOC is CK7 +, CK20 +/−, CDX2 +/−, PAX8 −, WT1 −, ER −, PR −, and SATB2 – [29].

## 4. Genomic Profile

Advancement in pathology and molecular data has allowed for consideration of MOC as a separate entity from other EOC subtypes. Cheasley et al. recently reported a comprehensive analysis of the MOC genetic profile in comparison to many histological types and proved that MOC is a genetically-unique entity [30]. Table 2 compares the frequency of molecular mutations in MOC, HGSC, and mucinous and non-mucinous CRC [6,11]. *KRAS* mutation is the most frequent molecular alteration in MOCs, with 46% having this mutation. While *TP53* mutation is typically associated with HGSC, about 25% of MOCs harbor this alteration as well. The amplification of *HER2* is also observed in 18% of MOCs. Moreover, high microsatellite instability (MSI-H) has been reported in MOCs [31]. Aberrant signaling in the wingless (WTN) pathway in the form of a mutation in *CTNNB1* or *APC* gene has also been documented. It is believed that the *KRAS* mutation develops as a first event, as the mutation is detected in the surrounding borderline and benign lesions, and *HER2* amplification or *TP53* mutation occurs at a later stage during malignant transformation, as this is observed exclusively in carcinomas [6,11,12].

To explore the molecular alterations in MOC, Friedlander et al. extensively evaluated the molecular profile of 304 cases of MOCs to investigate potential therapeutic targets. Alterations in MAP kinase pathway were the most common (49% mutations in *KRAS* and 3.5% in *BRAF*). mTOR pathway alterations were less likely (*PIK3CA* in 12% and *PTEN* in 6%). cMET overexpression was observed in 33% of cases, but no *cMET* gene amplification was seen. *p53* mutation was documented in 37% of cases and *EGFR* (epidermal growth factor receptor) gene amplification was seen in 50%. *HER2* gene amplification was found in 11% of cases. PD-1 positivity was detected in tumor-infiltrating lymphocytes in 43% of cases and PD-L1 was positive in 14% cases [32]. At the molecular level, MOC is a heterogeneous disease and its molecular landscape still poorly understood.

## 5. Work Up

Pre-operative assessment to investigate an adnexal mass or possible ovarian carcinoma includes a detailed history and examination, laboratory investigations, and radiological imaging. This allows for a narrowing of the differential diagnosis and hence management and appropriate counselling.

### 5.1. Tumor Markers

Tumor markers are a routine part of the work up of any suspicious adnexal mass. MOC is frequently linked with an elevated level of CA125, CEA, and CA19-9 [16]. Carcinoembryonic antigen (CEA) is the most valuable tumor marker to identify MOC pre-operatively. While CEA is elevated in one-third of all ovarian carcinoma, it is more likely to be elevated in MOC than non-mucinous ovarian carcinoma, 88% vs. 19%, respectively [33,34].

Free B-hCG might be infrequently overexpressed in epithelial ovarian carcinoma, including MOC [35]. In a pre-clinical module, overexpression of hCG was shown to induce tumor development, increase cell proliferation, induce cell cycle progression, and downregulate apoptosis [36]. Overexpression is associated with poor prognosis and unfavorable overall survival [37]. B-hCG can be used as a tumor marker to assess tumor response and recurrence. However, pregnancy should be ruled out in patients of reproductive age.

### 5.2. Magnetic Resonance Images (MRI)

A computed tomography (CT) scan is an essential part of evaluating the possibility of carcinoma. It aids in identifying the primary carcinoma and in evaluating the extent of disease. An MRI may provide additional information to differentiate primary MOC from metastatic mucinous carcinoma [38]. In MRI, an MOC tumor appears as a multilocular cystic lesion containing a solid part of intermediate intensity on T2-weighted MR images, hyperintense on diffusion-weighted images, and, with a type-3 enhancement curve, earlier enhancement relative to the myometrial curve. Extra-ovarian spread indicates malignancy [39].

Metastatic mucinous carcinoma from a colorectal origin has a stained glass appearance of a multilocular lesion with loculi on T1-weighted MR images of variable intensity. The amount of solid component differs and necrotic components that are hyperintense with T2-weighted MR images are often seen. Injection of gadolinium displays enhancement of the septa and the solid part when present. This appearance can be similar to MOC; in this case, Seidman criteria are useful to distinguish between diseases [40].

### 5.3. Gastrointestinal (GI) Investigation

As mentioned before, 80% of mucinous carcinoma of the ovary are metastatic. GI investigations, such as upper GI endoscopy and/or colonoscopy, have to be considered when evaluating such pathology. Some authors [12,23] suggest the following indications for proceeding to endoscopy/colonoscopy:

- Clinical or radiological findings suggesting a non-ovarian origin, based on tumor size, laterality, peritoneal spread, and advanced stage;
- CA 125 (KU/L)/CEA (ng/mL) ratio less than 26;
- Postoperatively, if pathological findings strongly suggest a GI primary.

## 6. Prognosis

Early-stage MOC has an excellent prognosis, with more than a 90% 5-year overall survival (OS), but survival in metastatic disease ranges between 12 and 30 months [12,41].

MOC subtypes carry slightly different prognoses even in early stage disease. The infiltrative subtype is linked to a higher risk of relapse, peritoneal spread, lymph nodes involvement, and mortality [12]. Gouy et al. reported on a retrospective analysis of stage I MOC according to histological subtypes. They included 64 patients, 29 with expansile and 35 with infiltrative subtypes. The 5-year OS and disease progression survival (DPS) were better in the expansile subtype, although not statistically significant (96% vs. 87% and 88% vs. 83%, respectively) [42]. The degree of differentiation had no prognostic value. Therefore, no grading is recommended for MOC [23]. The influence of capsule status in MOC on oncological outcome was evaluated by H. Kajiyama et al. They found a significant difference in OS and recurrent rate between stage IA, IC1, and IC2-3. The 5-year OS rates were 95.8%, 82.5%, and 82.9%, respectively. They concluded that capsule status was of significance [43].

Alexander et al. looked at the outcome of advanced MOC compared to serous epithelial carcinoma. They identified a reduced response to platinum-based chemotherapy, which reflected a worse progression-free survival (PFS) and OS (11.4 vs. 17.5 months and 21.6 vs. 47.2 months, respectively) [44]. Additionally, Mackey at al. published a meta-analysis of 7 trials, including 264 patients with MOC, comparing the outcome to serous epithelial carcinoma. OS was significantly worse in metastatic MOC (14.6 vs. 40.8 months) [45]. The difference in poor prognosis was not statistically significant between advanced MOC and metastatic mucinous carcinoma. The most important prognostic factor was the presence of residual disease at the end of cytoreductive surgery [26]. The inferior outcome in the advanced stage is also related to inadequate response to standard chemotherapy [6,11].

## 7. Surgical Management

The gold standard surgical management of all EOCs, including MOC, is a staging procedure for early disease and cytoreductive surgery for advanced disease [11,18,19,46]. Staging surgery includes peritoneal washing for cytology, hysterectomy, bilateral salpingo-oophorectomy, pelvic and para-aortic lymphadenectomy, omentectomy, and multiple peritoneal biopsies. In apparent confined disease, the risk of positive cytology and microscopic involvement of the omentum are 5.7% and 1.7%, respectively [12]. Cytoreductive surgery involves removing all measurable disease aiming for microscopic residual disease.

### 7.1. Routine Lymphadenectomy

Retroperitoneal lymph nodes assessment is part of the staging surgery in all EOCs [47]. It is understood that the chance of lymph node involvement in grossly confined non-mucinous epithelial carcinoma of the ovary is 20–30%. The role of routine systematic lymphadenectomy in early-stage MOC is, however, debatable.

Hoogendam et al. performed a systematic review and meta-analysis to investigate the role of routine lymphadenectomy for apparent stage I and II and the survival benefit of the procedure. They included 11 observational studies with 278 patients. The risk of lymph node involvement in apparent stage I and II disease when lymph node sampling or lymphadenectomy was performed was 0.8% and 1.2%, respectively. It therefore required 83 lymphadenectomies in apparent early-stage disease to detect one patient with a positive node. Furthermore, the authors could not prove any potential survival advantage. While keeping in mind that this meta-analysis involved small observational studies that were heterogeneous, the authors advised against performing lymphadenectomy for apparent early disease as the risks outweighs the benefits [48]. Schmeler et al. performed a retrospective study to assess the prevalence of lymph node involvement in early-stage MOC. The analysis included 107 patients, in which 51 underwent pelvic and para-aortic lymphadenectomy. They did not find any case of lymph node metastasis [49]. A retrospective observational study by Matsou et al. assessed the impact of lymphadenectomy on survival in early-stage EOC, including 4066 patients with mucinous histology, of which 2210 underwent a lymphadenectomy. Adequate lymphadenectomy enhanced survival in all subtypes except mucinous carcinomas [50].

Muyldermans et al. retrospectively looked at the risk of lymph node involvement in MOC according to histological subtypes. They included 44 patients, in which only 20 underwent lymphadenectomy. They confirmed a higher rate of lymph node metastasis in the infiltrative subtype (3 out 10), but no positive lymph node metastasis in the expansile subtype. They proposed that lymph node assessment can be omitted in the early-stage expansile subtype but must be performed in the infiltrative subtype [23]. M. Kleppe et al. reviewed 8 studies with 155 patients, but a revision of pathology was not performed. They found that the incidence of lymph node involvement in MOC stage I and II was 2.6% [51].

### 7.2. Appendectomy

Appendectomy has been recommended in all mucinous ovarian tumors. Lin et al. performed a retrospective analysis to assess the role of appendectomy in the surgical management of mucinous ovarian tumors. They concluded that the appendix should not be removed unless it is grossly abnormal, as there was no abnormal pathology found in the normal-appearing appendix [52]. A retrospective chart review by A. Cheng et al. assessed 164 patients based on the necessity of an appendectomy in MOC. Among 44 patients with MOC who had an appendectomy, there were 5 unusual looking appendixes with abnormal pathology and one normal-appearing appendix with microscopic involvement by primary MOC. They therefore recommended that careful intraoperative inspection of the appendix is mandatory, but a normal-looking appendix should not be routinely removed [53].

Rosendahl and colleagues evaluated the importance of appendectomy in the surgical staging of MOC. From the Danish Gynecologic Cancer Database, they retrieved data on 269 patients with MOC, of which 172 had an appendectomy. Pathological evaluation exhibited 10 cases of malignant involvement of the appendix (4%), of which 2 had a normal-looking appendix. However, all patients with appendicular involvement had other metastases and this finding did not change the stage. They strongly advised for performing appendectomy for all suspected cases of MOC since a normal-appearing appendix does not exclude metastasis. Additionally, appendectomy was not associated with increased morbidity [54].

Some gynecologic oncologists are still advocating for routine appendectomy as part of surgery for MOC. It is challenging at times to distinguish a primary MOC from metastatic mucinous carcinoma and removal of the appendix might help with the deferential diagnosis and improve staging.

### 7.3. Fertility Sparing Staging (FSS)

MOC is the most prevalent epithelial carcinoma among women of reproductive age [8] with 80% of all MOC presenting as stage I [10]. FSS is frequently addressed during the management of such pathology. Gouy et al. looked at the outcome of stage I MOC after FSS, according to histological subtype (expansile and infiltrative). They retrospectively evaluated 21 patients, 12 expansile and 9 infiltrative subtypes. The histological subtype did not influence the oncological outcome. They concluded that FSS is safe for stage I MOC with an excellent prognosis but should not be offered for stage IC3 and beyond [55]. However, Rodriguez et al. reported a worse survival with more relapses with the infiltrative subtype when they evaluated 26 patients with MOC [56]. Bentivegna et al. reported the long-term outcome of over 500 EOC patients treated with FFS. The most common subtype was MOC, with 280 patients. The recurrence rate was 6.8%. The majority recurred as extra-ovarian disease with it being lethal in 63% of patients [57]. It is debatable whether the recurrence was because of preserving the ovary or because of the nature of the disease. The role of FSS should therefore be adequately discussed in these patients.

### 7.4. Hyperthermic Intraperitoneal Chemotherapy (HIPEC)

HIPEC has been a therapeutic option to treat peritoneal carcinomatosis from different primary sites. The role of HIPEC in managing EOCs has been emerging in recent years. A recent phase III multicenter randomized control trial by Van Driel et al. exhibited improvement in OS and PFS when HIPEC, using cisplatin 100 mg/m^2^, was added to the standard interval debulking surgery (IDS) in stage III EOC. There was no increased rate of perioperative complications in the HIPEC arm. The majority of patients had the HGSC subtype, with only 3 patients having MOC [58]. This publication resulted in a change in the National Comprehensive Cancer Network (NCCN) 2019 guideline to include HIPEC at the time of IDS as a level 2A recommendation. Two meta-analyses also showed a superior survival advantage among EOC treated with HIPEC in both the primary and recurrent settings [59,60]. However, a large multicenter retrospective analysis on the benefit of HIPEC with cytoreductive surgery in rare ovarian cancer subtypes showed no therapeutic benefit in the MOC subtype [61]. Multiple trials are ongoing or have completed recruitment to further investigate the benefit of HIPEC in managing both primary and recurrent EOC [59,62].

HIPEC is a recognizable treatment modality in managing metastatic CRC and pseudomyxoma peritonei. It has improved survival when combined with complete cytoreductive surgery [63,64,65]. Understanding the behavioral and biological similarities between MOC, CRC, and pseudomyxoma peritonei, HIPEC seems to be a reasonable option in treating metastatic MOC. However, controversy remains as to what chemotherapeutic agent, gynecological or colorectal, should be used. Due to the very low incidence of MOC, it is challenging to have specific data on HIPEC for MOC, although data may be extrapolated from EOC and CRC trials.

## 8. Chemotherapy

The current standard of care in managing all EOCs, including MOC, is surgical staging for early disease and cytoreductive surgery for advanced stage disease followed by platinum-based chemotherapy [11,12]. The most frequently used regimen in MOC is the doublet of carboplatin and paclitaxel, which is the standard protocol for all EOCs. The landmark practice-changing clinical trials in EOCs included a small percentage of MOC (2.5%–7%) [66,67,68,69]. Because of the low prevalence of MOC, clinical trials primarily related to MOC are lacking.

While adjuvant chemotherapy reduces the risk of recurrence in early-stage HGSC, the benefit is not clear in early-stage MOC. The two main trials in adjuvant chemotherapy in early-stage EOC, ACTION, and ICON-1, included 180 patients with MOC and did not show a statistically significant reduction in the recurrence rate between the observation arm and the treatment arm [70,71]. Nasioudis et al. retrospectively analyzed the data of 4242 patients, retrieved from the National Cancer Data Base (NCDB) in the United States, to explore the benefit of chemotherapy in early-stage MOC. There was no statistically significant difference in 5-year OS between patients who did or did not received chemotherapy in stage 1A/1B and 1C, which were 86.8%, and 89.7%, respectively. The difference remained the same, even after stratification by disease sub-stage and tumor grade. The researchers concluded that, as evidence is scarce, offering adjuvant chemotherapy in this setting should be individualized and discussed with patients [72].

MOC has been shown to be less responsive to platinum-based chemotherapy compared to other EOC subtypes. The response to the standard chemotherapy regimen is reflective in the overall outcome. Several investigators have confirmed MOC to be platinum-resistant. Response rates were between 12% and 35% in MOC compared to 70% in HGSC [17,73].

Due to the biological and molecular similarities of MOC and mucinous CRC, GI chemotherapy protocols have been proposed as an alternative to the standard gynecology regimen. The various GI protocols and their evidence in MOC are summarized in Table 3. The Gynecology Oncology Group (GOG) trial 241 was designed to explore the benefit of a colorectal regimen in newly diagnosed MOC. It was a phase III trial that randomly assigned patients to carboplatin/paclitaxel or capecitabine/oxaliplatin. There was a second randomization to bevacizumab or placebo to assess the activity of this antiangiogenic agent. Unfortunately, due to slow accrual, the trial was only able to recruit 50 women and was prematurely terminated. Data from the recruited patients showed no statistically significant difference in progression-free survival and toxicity profiles between the treatment arms. A central pathology review was performed on 40 of 50 cases, with only 45% confirmed to be primary MOC, with the rest being metastatic mucinous carcinoma [74]. While the bevacizumab arms did not have a statistically significant superior PFS, it was hard to draw any conclusions due to the small number of participants. Acknowledging the low response rate of MOC to standard therapy and understanding the molecular profile of this carcinoma, targeted therapy may be potentially beneficial.

**Figure 1:**
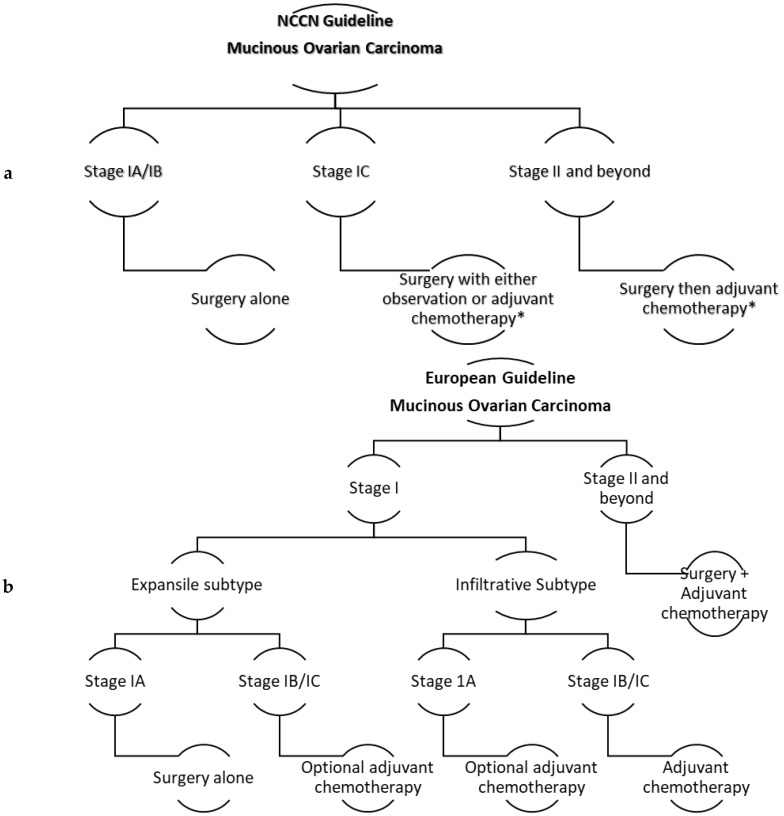
(**a**) The NCCN guideline for managing MOC. * Adjuvant chemotherapy is platinum-based, either carboplatin/paclitaxel or oxaliplatin with fluorouracil or capecitabine. (**b**) The European guidelines for managing MOC. * Adjuvant chemotherapy options are carboplatin/paclitaxel, xaliplatin with 5-FU, or capecitabine (anecdotally preferred). * Consider bevacizumab for either regimens (no clear evidence). * Consider neoadjuvant chemotherapy for unresectable biopsy-proven disease.

Figure 1 displays the National Comprehensive Cancer Network (NCCN) and the European guidelines in managing MOC [83].

## 9. Targeted Therapy

The recently proven efficacy of PARPIs (poly adenosine diphosphate-ribose polymerase inhibitors) in managing non-mucinous type EOC is a milestone in ovarian cancer management. It is the first targeted agent to be approved in ovarian cancer treatment in both the primary and recurrent settings. Unfortunately, PARPIs have no role in the management of MOC as these tumors are not associated with *BRCA* mutations or homologous recombinant deficiency.

### 9.1. VEGF Inhibitor

The vascular endothelial growth factor (VEGF) inhibitor, bevacizumab, has been shown to improve PFS in EOC in the primary setting and in the platinum-sensitive and platinum-resistant recurrence setting. Notably, subgroup analyses revealed that adjuvant bevacizumab improves OS in sub-optimally cytoreduced disease. All phase III bevacizumab EOC trials included a small number of MOCs [84,85,86,87]. It has also exhibited improvement in OS and PFS in metastatic colorectal carcinoma in multiple trials and meta-analyses [88,89].

### 9.2. EGFR Monoclonal Antibodies

Cetuximab use results in enhanced response rate and duration of response in the first-line treatment of metastatic CRC in patients where there is EGFR expression and where the tumor is *KRAS* wild-type [90]. The results of cetuximab on ovarian cancer as a single therapy or in combination with the standard chemotherapy were disappointing [91,92]. However, these phase II trials included all EOC subtypes without specification of *KRAS* status. Sato et al., in a preclinical study, reported that cetuximab was only able to employ anti-proliferative activity in MOC cell lines, which did not have *KRAS* mutations [93]. Hence, the value of anti-EGFR monoclonal antibody therapy may be limited to *KRAS* wild-type cases. Further data is required on the effectiveness of cetuximab on *KRAS* wild-type MOC.

### 9.3. Anti-HER2 Therapy

Anti-HER2 therapy has been proven to be successful in managing *HER2*-amplified breast cancer [94]. Trastuzumab, a *HER2* monoclonal antibody, showed benefits to OS in *HER2*-positive gastric carcinoma [95]. McAlpine et al. reported the anecdotal efficacy of trastuzumab in combination with chemotherapy in 2 out of 3 patients with MOC who had *HER2* amplification [96]. Jain et al. published a single case of successful treatment of progressive *HER2*-positive MOC with trastuzumab and lapatinib [97]. Nevertheless, more data on anti-HER2 therapy in *HER2*-amplified MOC is needed.

## 10. Conclusions and Future Direction

MOC is a distinct disease among the EOC subtypes and it is different from GI mucinous carcinoma. This is evident based on its clinical behavior, pathological features, molecular profile, prognosis, and response to the standard treatment. The true incidence of MOC is 3% of all EOCs and it is the most common subtype among young women. Up to 80% of MOCs present as early stage disease, which carries an excellent prognosis. Unfortunately, advanced stage disease is associated with a much poorer prognosis compared to HGSC because of the low response to platinum-based chemotherapy.

However, it is still challenging for pathologists to make a diagnosis of MOC, with up to 60% having metastatic mucinous carcinoma, after central pathology review [42]. Differentiation of primary MOC from metastatic mucinous carcinoma demands cautious microscopic examination. The clinical picture created with Seidman criteria and the IHC profile should help pathologists in reaching an accurate diagnosis. Additionally, the identification of invasive components in an otherwise borderline mucinous tumor requires vigilant assessment. These tumors are usually very large and require judicious sampling.

Some aspects of the surgical management of MOC are still uncertain. Based on the current data, routine pelvic and para-aortic lymphadenectomy can be omitted in grossly confined expansile-type MOC but should be performed in infiltrative type. The necessity of performing an appendectomy is uncertain at present. FFS is an option in young, selected patients after proper counseling. In advanced disease, cytoreductive surgery and the amount of residual disease at the end of the surgery are the most critical factors in prognosis.

While it is known that MOC is chemotherapy-resistant or that it does not respond to the standard chemotherapy regimens, there are no better treatment options available at present. To date, there have been no successful prospective phase II or III randomized clinical trials directed specifically to MOC. Failure of GOG 241 to achieve the targeted accrual is a clear example of how challenging it is to obtain high-level data on rare tumors.

MOCs harbor a variety of molecular alterations that open the door for a potential benefit of a molecularly guided approach. *KRAS* mutation and *HER2* amplification are frequent in MOC and are mutually exclusive. Early data of cetuximab and trastuzumab are promising, but other alterations could also be targeted. Platinum-based chemotherapy can be considered for MOC positive *TP53* mutations. Kommoss et al. suggested three subdivisions of MOC based on molecular subtypes, namely: *HER2* over amplification, *KRAS* mutations, and tumors with neither KRAS nor HER2 abnormalities [11]. This categorization may forge new prospects in influencing treatment decisions and novel targeted therapy advancement in this rare type of EOC. Lastly, a project similar to the Genomic Atlas project focusing on MOCs may well be helpful in the understanding and treatment of this disease.

## Figures and Tables

**Table 1:** Summary of the IHC expression of MOC and metastatic mucinous carcinoma.

	MOC Intestinal Type	MOC Endocervical Type	CRC	Pancreatic	Biliary	Gastric	Cervical
CK7	+	+	−	+/−	+/−	+/−	+
CK20	+/−	−	+	−/+	−/+	−/+	−/+
CDX2	+/−	−	+	+/−	+/-	+/−	−/+
CEA	+/−	−	+	+/−	+/-	+/−	+/−
CA 125	−	+	−	+/−	+/-	−	+
CA 19-9	+	−/+	+	+	+	+	-
ER	−	+	−	−	−	−	−/+
DPC4	+	+	+	+ or −	+ or −	+	+
P16	−	−	−/+	−	-	−	+

MOC: Mucinous Ovarian carcinoma; CRC: Colorectal carcinoma; +: diffusely positive; −: diffusely negative; +/−: diffusely positive or focally negative; −/+: diffusely negative or focally positive.

**Table 2:** Frequency of molecular alterations in MOC, HGSC, mucinous, and non-mucinous CRC.

Molecular Alteration	MOC	HGSC	Mucinous CRC	Non-Mucinous CRC
*KRAS* mutation	33–46%	10–22%	31–48%	24–33%
*BRAF* mutation	0–9%	0%	15–27%	6–12%
*TP53* mutation	26–55%	96%	31–41%	41%
*HER2* amplification	18–35%	-	<1%	2%
MSI-H	22%	13.8%	25–36%	3–6%
*APC/CTNNB1* mutation	9%	-	24%	88%

MOC: mucinous ovarian carcinoma; HGSC; high-grade serous carcinoma; CRC: colorectal carcinoma; MSI-H: high microsatellite instability.

**Table 3:** Summary of the GI protocols used in MOC.

Chemotherapy Regimen	Response Rate	Remarks
FOLFOX BCCA protocol: Oxaliplatin 85 mg/m^2^, IV over 2 h. Leucovorin 400 mg/m^2^, IV over 2 h. 5-FU 400 mg/m^2^, IV push after LV, then 5-FU 2400 mg/m^2^, IV infusion over 46 h. The cycle is repeated every 2 weeks.	About 30%	Evidence on heavily pretreated EOC. A limited number of MOC in the studies. Prospective phase II [75,76]. Retrospective reviews [77,78]. Various slightly different doses have been tested.
XELOX BCCA protocol: Day 1: Oxaliplatin 130 mg/m^2^, IV. Day 1–14: Capecitabine 1000 mg/m^2^, orally twice per day. The cycle is repeated every 3 weeks.	No data in ovarian cancer.	High response rates were seen in colorectal cancer [79,80]. Capecitabine is the oral analog of 5-FU. Single-agent 5-FU showed modest response in pretreated advanced EOC [81]. Single-agent Oxaliplatin demonstrated some activity in pretreated EOC in phase II trial [82].

5-FU: 5-fluorouracil; EOC: Epithelial ovarian carcinoma; MOC: Mucinous ovarian carcinoma; h: hours; LV: Leucovorin; BCC: British Columbia cancer agency; IV: intravenous.
